# Women’s autonomy and maternal health decision making in Kenya: implications for service delivery reform - a qualitative study

**DOI:** 10.1186/s12905-024-02965-9

**Published:** 2024-03-19

**Authors:** Easter Olwanda, Kennedy Opondo, Dorothy Oluoch, Kevin Croke, Justinah Maluni, Joyline Jepkosgei, Jacinta Nzinga

**Affiliations:** 1 KEMRI-Wellcome Trust Research Programme Nairobi, Nairobi, Kenya; 2 Department of Global Health and Population, Harvard TH Chan School of Public Health, 677 Huntington Ave, Boston, MA 02115 USA

**Keywords:** Redesign, Autonomy, Agency, Women, Empowerment, Maternal, Child, Health

## Abstract

**Background:**

Maternal and neonatal outcomes in, Kakamega County is characterized by a maternal mortality rate of 316 per 100,000 live births and a neonatal mortality rate of 19 per 1,000 live births. In 2018, approximately 70,000 births occurred in the county, with 35% at home, 28% in primary care facilities, and 37% in hospitals. A maternal and child health service delivery redesign (SDR) that aims to reorganize maternal and newborn health services is being implemented in Kakamega County in Kenya to improve the progress of these indicators. Research has shown that women’s ability to make decisions (voice, agency, and autonomy) is critical for gender equality, empowerment and an important determinant of access and utilization. As part of the Kakamega SDR process evaluation, this study sought to understand women’s processes of decision-making in seeking maternal health care and how these affect women’s ability to access and use antenatal, delivery, and post-natal services.

**Methods:**

We adapted the International Centre for Research on Women (ICRW) conceptual framework for reproductive empowerment to focus on the interrelated concepts of “female autonomy”, and “women’s agency” with the latter incorporating ‘voice’, ‘choice’ and ‘power’. Our adaptation did not consider the influence of sexual relationships and leadership on SRH decision-making. We conducted key informant interviews, in-depth interviews, small group interviews and focus group discussions with pregnant women attending Antenatal clinics, women who had delivered, women attending post-natal clinics, and men in Kakamega County. A thematic analysis approach was used to analyze the data in NVivo 12.

**Results:**

The results revealed notable findings across three dimensions of agency. Women with previous birthing experiences, high self-esteem, and support from their social networks exhibited greater agency. Additionally, positive previous birthing experiences were associated with increased confidence in making reproductive health choices. Women who had control over financial resources and experienced respectful communication with their partners exhibited higher levels of agency within their households. Distance relational agency demonstrated the impact of health system factors and socio-cultural norms on women’s agency and autonomy. Finally, women who faced barriers such as long waiting times or limited staff availability experienced reduced agency in seeking healthcare.

**Conclusions:**

Individual agency, immediate relational agency, and distance relational agency all play crucial roles in shaping women’s decision-making power and control over their utilization of maternal health services. This study offers valuable insights that can guide the ongoing implementation of an innovative service delivery redesign model, emphasizing the critical need for developing context-specific strategies to promote women’s voices for sustained use.

**Supplementary Information:**

The online version contains supplementary material available at 10.1186/s12905-024-02965-9.

## Background

Globally, the provision of health care is evolving towards providing care that is respectful of and responsive to individual patient preferences, needs, and values, and ensuring that patient values guide all clinical decisions [1]. Patient-centered care, in relation to clinical decision-making, is grounded in concepts of intrinsic values, personal preferences, and partnership [2]. A patient-centered approach, empowers women by involving them in decision-making processes and respecting their choices [3]. Women’s empowerment, in turn, enhances their autonomy and ability to seek appropriate and timely maternal health care [4], ultimately affording women agency for their overall well-being. When women are not empowered or lack autonomy, they may face barriers to accessing quality care, resulting in adverse maternal health outcomes [5].

Research on patient-centered care for women shows that women’s autonomy (i.e., the freedom of women to exercise their judgment in order to act for their own interests) influences reproductive, maternal, and child health outcomes [6–11]. Conversely, the absence of women’s autonomy in decision-making results in delays and poor utilization of maternal health services and ultimately increased maternal morbidity and mortality [12]. Thus, empowered women can make informed decisions about their reproductive health, including family planning, timing and spacing of pregnancies, and the type of care they receive during pregnancy and childbirth [13–16].

Freedom of mobility, participation in household decision-making, and self-efficacy are key dimensions of women’s empowerment [17, 18]. Empowering women to make their own decisions, pursue goals, and control their lives and resources is a crucial aspect of Sustainable Development Goal (SDG) 5, which seeks to attain gender equality and empower all women and girls [19]. Moreover, empowering women in reproductive and sexual matters is crucial, as intimate relationships often involve significant power imbalances that can limit women’s ability to negotiate with their partners on sexual issues [20].

The International Centre for Research on Women (ICRW) has developed a conceptual framework for reproductive empowerment merging “female autonomy” and “women’s agency”, which includes ‘voice’, ‘choice’, and ‘power’. This framework is relevant to women’s autonomy in Kakamega as it provides a comprehensive approach to understanding the factors influencing women’s agency and empowerment, informing targeted interventions and policies in the region. Women’s individual agency involves expressing opinions, making decisions about their lives, and pursuing their aspirations, which empowers them to assert their voices and seek personal and professional growth. Immediate relational agency focuses on the influence of close relationships such as family, friends, and intimate partners on an individual’s agency [21]. These relationships can either support or restrict a woman’s ability to exercise her voice, make choices, and pursue empowerment. Supportive relationships enable women to exercise their agency freely, while oppressive ones may limit their ability to make decisions and assert their voice, hindering empowerment. Distant relational agency refers to the broader social, cultural, and institutional influences that shape women’s agency and empowerment [22]. Societies that prioritize gender equality, offer legal protections, ensure access to education and healthcare, and promote women’s participation in decision-making enhance women’s agency. Conversely, gender inequalities and limited opportunities hinder women’s agency and empowerment.

**Fig. 1:**
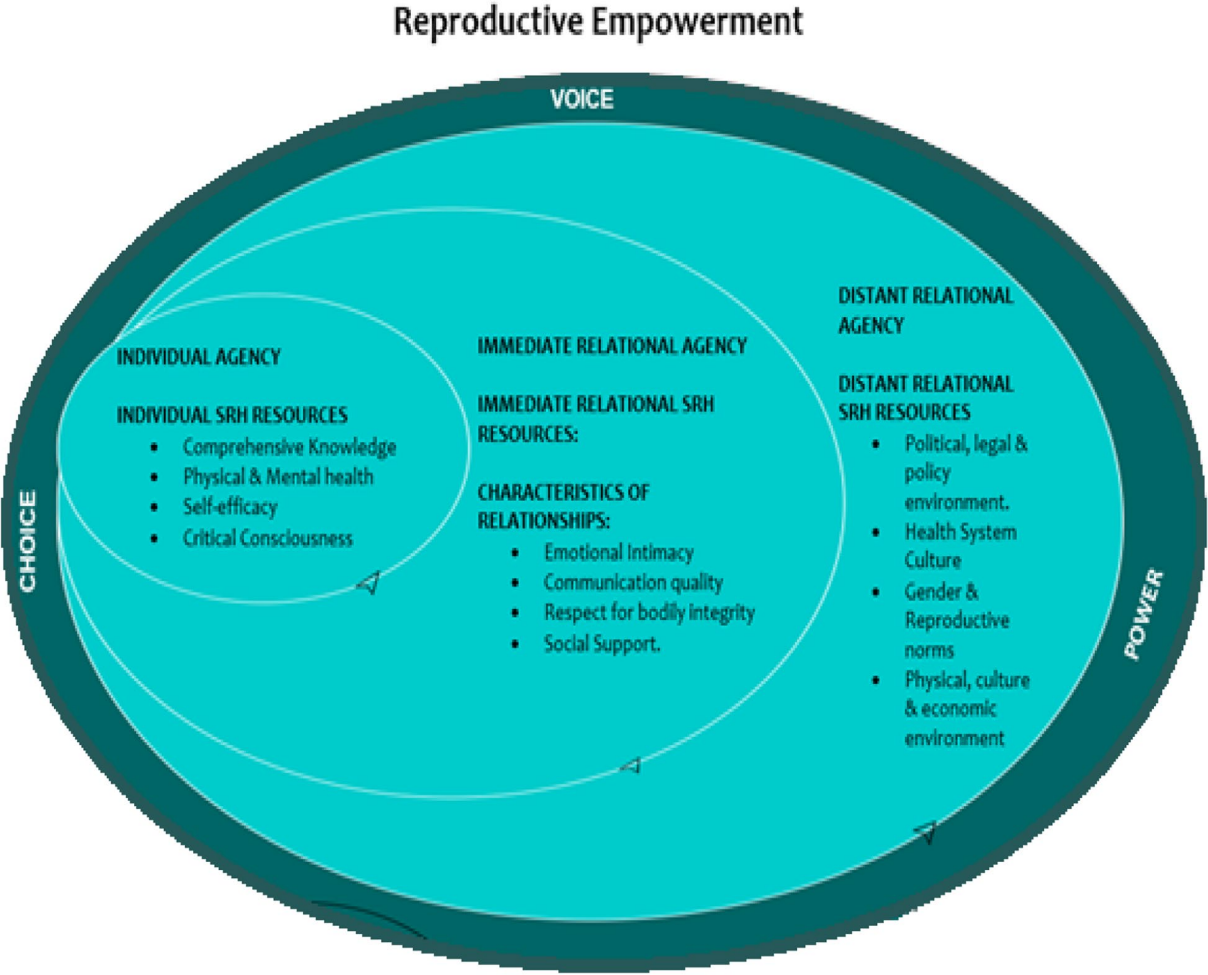
Borrowed from the Conceptual Framework of Reproductive Empowerment by the International Center for Research on Women (ICRW)

The relationship between individual, immediate, and distant relational agency is complex and interconnected, with individual agency influenced by both immediate and distant relational agency. Supportive immediate relationships and equitable social structures can enhance individual agency, enabling women to exercise their voice and make choices while repressive immediate relationships and restrictive social structures can limit women’s agency [23]. Aligning all three levels of agency positively leads to women’s voice, choice, power, and empowerment. These concepts are illustrated in Fig. 1 below.

Evidence suggests that increasing women’s mobility can empower them to exercise greater control over their lives by increasing their access to healthcare, education, markets and information [6, 24]. Additionally, women with strong sense of self-efficacy have the potential to anticipate different success scenarios, persevere in the face of obstacles, take action against the existing social norms [25], and navigate complicated healthcare contexts to receive care [26]. Relative to maternal and child health, increased postpartum maternal self-efficacy been has associated with improved functional status in the postpartum period [27]. Furthermore, women’s control over resources and decision-making within a household plays a crucial role in enhancing healthcare-seeking behaviors and maternal and child health outcomes [28]. Changes in women’s intra-household bargaining power, also increases a woman’s status and impacts her decision-making ability [29, 30]. All these are key considerations in determining the implementation process and success of maternal and child health interventions and reforms. Existing literature primarily emphasizes the impact of women’s choice and agency on health decision-making and service use, but there is limited documentation on how women’s autonomy experiences can inform the implementation of improved maternal and child health interventions.

### The implementation context

Kakamega County was selected due to its high maternal mortality rate of 316 per 100,000 live births and neonatal mortality rate of 19 per 1,000 live births, alongside approximately 70,000 births in 2018, distributed with 35% at home, 28% in primary care facilities, and 37% in hospitals. Part of recommendations from the Lancet Global Health Commission on High Quality Health Systems for improving quality of care is the implementation of “Service Delivery Redesign for Maternal and Newborn Health”, (SDR) reform. Kakamega County started the phased implemention of SDR in 2021 and is currently in its improvement phase.

During the SDR implementation period, improvements were made to health facilities, including the commissioning of a new Maternity wing and an increase in bed capacity at Malava. The Linda Mama health scheme provides affordable maternal and child health services, and Malava sub-county hospital increased its claims from 34 to 79%. Additionally, a newborn unit was constructed, and the facility currently has a resident pediatrician, surgeon and gynecologist supported by nurses, clinicians, and medical officers.

Similarly, Lumakanda sub-county hospital has reorganized its infrastructure at the maternity wing and the newborn unit, introduced emergency evacuation services, implemented pseudo-facility improvement fund (FIF) disbursements, enhanced accountability and visibility of blood resources through a blood tracker dashboard, enrolled women for pregnancy care text prompts, and provided Emergency obstetric and newborn care (EmONC) training to its staff while also training health workers from primary health care facilities. A summary of the SDR related implementation activities can be found in the Supplementary file 1.

This paper aimed at examining how women’s processes of decision-making in seeking maternal and neonatal health care both influences and is influenced by the implementation of SDR in Kakamega county. It is anticipated that the learning from this paper will inform the implementation of SDR by highlighting patient voice in reforming delivery of MCH services.

## Methods

### Study setting

The study took place in Malava and Lumakanda sub-County hospitals which are maternity centers of excellence where the Kakamega Service Delivery Redesign (SDR) is being implemented. The Kakamega maternal and child health Service Delivery Redesign (SDR) is a structural reform that aims to reorganize the maternal and newborn health services by shifting deliveries for all women to advanced facilities that offer definitive care for complications. The reform is now being implemented by the Kakamega County government with the aim of improving the quality of antenatal, delivery and postnatal care. It purposes to ensure that all women can give birth in safe environments, with skilled providers that have the tools and competencies to care for women during uncomplicated birth and who can detect and deal with complications if and when they occur [31].

### Study design

This was a cross-sectional exploratory study using qualitative research methods with purposively selected participants. We conducted key informant interviews, in-depth interviews, small group interviews and focus group discussions with pregnant women attending Antenatal clinics, women who had delivered, women attending post-natal clinics, and men in Kakamega County. In this context, small group interviews involved a small group of individuals, allowing for in-depth exploration of individual perspectives within a group setting, while focus group discussions were conversations guided by a moderator, emphasizing interactions among 8–10 participants to explore shared perspectives and group dynamics.

The FGDs had an average of 8 individuals while the small group discussions had an average of 4 individuals. For the FGDs demographic dynamics such as gender and age were managed through the selection of participants to ensure diverse representation within the groups. We formed homogeneous groups based on specific demographic criteria, to foster open and comfortable discussions among participants with shared characteristics. In this case we had FGDs specifically for younger women, another for older women, and lastly with the men separately. Conversely, for small groups, we intentionally chose participants representing various demographic profiles to capture a range of perspectives and experiences. The FGDs lasted between 45 and 60 min, the IDIs lasted between 30- 45 min and the KII lasted an average of 45 min.

See the Table 1 below.

**Table 1:** Table of participants

	**Number**	**Respondents**
**FGDs**	2	Men and Birth companions
**IDIs**	19	5 ANC, 5 Delivery and 7 PNC clients, 1 birth companion, 1 CHV
**KII**	5	Medical superintendent, CHEW, TBA, Nursing officer in charge and Frontline MCH worker
**Small group interviews**	1	ANC in charge, PMTCT in charge, MCH in charge and HRIO

The pre-set inclusion criteria included willingness to consent and to participate in the study, and good knowledge or understanding of the areas of inquiry. We employed purposeful participant selection to include diverse demographic characteristics, experiences, and perspectives in relation to the research topic.

### Data collection

Data collection took place in November 2022 and in March 2023. Participants were recruited from the antenatal, delivery and post-natal clinics in Lumakanda and Malava sub-County hospitals. We conducted key informant interviews, in-depth interviews, small group interviews and focus group discussions with pregnant women attending Antenatal clinics, women who had delivered, women attending post-natal clinics, and men using guides with questions developed in English and then translated into Swahili.

The interviews covered three broad topics of interest (1) factors that affect women’s autonomy and decision-making power in the household, (2) the process of decision-making at the family level in seeking maternal health care during pregnancy, delivery, and the postpartum period, and (3) how the decision-making process affects women’s ability to access and use maternal health services. Interviews were audio-recorded for participants who consented. During the interviews, detailed descriptive field notes were written covering interactions between the interviewer and respondent, non-verbal communication, environment, and reflections from interview content. All interviews were transcribed verbatim in Swahili and translated to English then cross-checked to ensure appropriate data and its quality before data analysis. Interviews were conducted to the point of theoretical saturation through iterative data analysis of emerging themes which was done alongside data collection. Iterating between data collection and analysis enabled the research team to be mindful of their own biases and actively worked to mitigate them, thus contributing to the robust representation of opinions. A total of 27 interviews including the IDIs, KIIs and FGDs lasting 30-60 min were conducted sequentially.

### Data analysis

A thematic analysis approach was adopted. The English transcripts were read several times to develop familiarity with the raw data. Open coding was done to identify women’s expressions highlighting their autonomy; axial coding then followed to relate and label codes with shared concepts, dimension, and properties. Finally selective coding was done to delimit coding to the identified core concepts from the data [32]. See Table 2 below. E.O independently coded the data in the first phase of analysis. This was then followed by discussions between E.O and J.N, comparing emerging codes and developing a consensus on a final coding framework that was used to code and analyze the data in NVIVO 12.

**Table 2:** Selective coding table

	**Selective codes**	**Axial codes**	**Open codes**
1	Individual agency	Power Within	Fear
			Self-stigma
			Trust in self
			Social networks
			Previous birthing experiences
		Health Literacy/Awareness	Health seeking behavior
			Health education/sensitization/outreaches
			Media exposure
2	Immediate relational agency	Household decision making	Source of income
			Control of household finances
			Direct and indirect medical costs incurred
			Respect between couples
			Spousal communication
		Freedom of movement	Household structure
			Domestic/household chores
			Mistrust from spouses
3	Distance relational agency	Health system factors	Facility workload
			Facility wait time
			Staff availability
			Distance to the health facility
		Socio-cultural factors	Religious norms
			Male dominance
			Male involvement

## Results

This section presents the findings from an in-depth exploration of women’s agency and autonomy. Several key themes and sub-themes emerged, providing valuable insights into the complex dynamics surrounding women’s agency and autonomy. These findings contribute to a deeper understanding of the challenges and opportunities women face in asserting their rights, making choices, and navigating their social and cultural environments. The results further reveal the multifaceted nature of women’s agency and autonomy, encompassing individual, immediate relational, and distance relational factors and shed light on the interplay between these factors and uptake and utilization of maternal and child health services.

### Individual agency

Independent women with strong self-trust were more likely to exercise their agency with greater adaptability and confidence in decision-making. Such strong inherent trust mediated power within which consequently shaped women’s individual agency and their ability to make decisions regarding maternal care.

*“In my life, I’ve always been very independent and doing things on my own. I’ve never really encountered those challenges where I was told that someone else had to speak for me. Most of the time, I just do my own thing.”-ANC2*

Conversely, the perception of shame, and insignificance among young pregnant girls diminished their agency in seeking services.

*“For some, some are just afraid, especially those of young age. They are scared of coming to the clinic, so they try to hide the pregnancy because it brings them shame.” FGD men*

The power within was also influenced by other factors including social support and past experiences. Supportive social networks provided women with the necessary resources, information, and support to develop their power within and exercise agency. Our findings indicate that social networks played a crucial role in shaping women’s individual agency in maternal healthcare decisions as they provided women with information, knowledge, and resources that enhanced their agency. Women sought input from their social networks including, family members, friends, community health volunteers (CHVs), and birth companions. Some elements such as finances, relational trust, and experiences of people were considered before a social network was chosen to aid with the decision-making.

*“At times, the husband will help in decision making because it could be a place that is far and needs transport, he will be the one to decide whether I will go to Malava or not. The decision comes from him.”-PNC 1*

The contacts drawn from social networks also shared their previous birthing experience with women fostering a sense of agency. Negative birth experiences raised awareness of potential challenges and helped women make more informed decisions while choosing the best options for the next birthing experience. In such instances, while the objective was to look for quality, the previous experiences of others as well as social relationships with health workers constituted the yardstick in making the final choice as the experiences of some participants revealed:

*“That time I heard negative views about this place, and I didn’t feel the need to come. I heard that in Lumakanda they are very harsh and I told myself that “let me get used to Turbo”, I never set foot here. But in 2014, I said no, let me try the local facility.”-PNC 3*

*“Number one, etiquette of these nurses towards these pregnant women. You know you can go somewhere else, and the nurse just shouts at you. It’s like she carries all her stress to the hospital. But here in Lumakanda, I have not faced anything. So, that’s why I decided to come all the way.”-ANC 2*

Additionally, health literacy had a great influence on women’s individual agency through the knowledge, attitudes, and beliefs acquired through health education and media exposure. Collectively, we saw how health education, media exposure, and health-seeking behavior significantly impacted women’s individual agency and decision-making for maternal care. With improved knowledge, women were more empowered to actively participate in decision-making processes, assert their preferences, and access appropriate healthcare services. Of note, is how health and financial literacy empowered women by increasing their understanding and enabling them to actively participate in decision-making processes.

*“Maybe the media can broadcast that, mothers with children, and pregnant women, need to go to the hospital to get health services. They need to understand that they will benefit from going there. They need to hear it for themselves.”-PNC 3*

Additionally,* m*edia exposure had a substantial impact on shaping women’s perceptions and attitudes toward maternal care. Our findings show that access to accurate and reliable information through various media channels can contribute to informed decision-making and empower women to seek appropriate maternal care. The respondents identified and recognized the value of health literacy in gaining autonomy and control over their health and well-being as corroborated in the quote below.

*“Through media, newspapers, radios, councils, through these open places, many mothers have now discovered their rights and they have power now”-CHV.*

### Immediate relational agency

We found that women’s immediate relationships were also linked to household decision-making and freedom of movement. Our findings demonstrated that when women had their own source of income, it often led to increased empowerment and decision-making power within the household. Conversely, women who did not participate in income generation could hardly participate in decision-making in the house as recounted by this respondent.

*“The only thing affecting my ability to make decisions, it’s because I’m not working. I do see those who are working, have the ability to decide, they can do their own things, as they wish. Even if there are obstacles from the husband, she can decide, let me do this. But for someone like me, it’s difficult.”-PNC 3*

Health-related decisions, including seeking medical treatment, had financial implications. The costs associated with treatment impacted decision-making, particularly if they were substantial or necessitated long-term financial obligations. In such cases, decisions were made, considering the financial impact and available resources.

*“Our clients depend on their husbands and their mothers-in-law as decision-makers. Since they depend on them especially the husbands to give them transport to go to the facility, they (husbands) are the key decision makers on where they seek their service.”-NO-IC*

Spousal communication allowed for open dialogue, negotiation, and compromise, leading to decisions that consider the needs and preferences of both partners. Couples who trusted each other navigated power over each other by considering each other and providing a morale boost that facilitated decision-making in the household.

*“It’s my husband whom I confide in the most. I feel comfortable talking to him, especially during my pregnancy, when I had a lot of complications like high blood pressure, and even had to undergo an operation. He’s my husband, so I talk to him, and he’s the one who helps me.”-PNC 3*

Freedom of movement also influenced women’s immediate rational agency. Household structures were mostly patriarchal, where men held dominant roles and women’s mobility and decision-making power was limited. The traditional role of men as heads of households mandated a restricted life for women as they were expected to be submissive and show respect towards male partners.

*“You find in some cultures a woman cannot say anything in front of the husband. The man is the one who speaks. In some areas, you are told “When you get here you follow what the husband, the mother or the father says”. -ANC 1*

In contrast, single/separated women had complete freedom of movement and were able to decide alone. This was evidenced by one of the respondents, who shared that.

*“When it comes to decision-making, I don’t think anyone can influence me because I make decisions myself. Even if someone discourages me, I know what I’m doing and what I’m looking for.”-ANC 2*

Engaging in domestic tasks restricted women’s mobility outside the home. The demands of household chores meant that women could not move freely. Their social functioning was largely restricted to the household, the fields, and tending to children.

*“Well, there are hindrances, at times I am alone at home without a house help, and I need to go somewhere. There are cows to take care of and children who may have gone to school waiting for me to make lunch. You see, there are hindrances, and you cannot just leave like that until you plan yourself.”-PNC 3*

Mistrust within the household also undermined women’s freedom of movement and decision-making. These restrictions were reinforced by societal expectations and gender norms that prescribe women’s roles as primarily confined to the private sphere. Women’s ability to move about was sometimes constrained due to trust issues between spouses.

*“You know, sometimes I come here in the morning and leave at two or three. He thinks it’s not just the hospital, he doesn’t understand, but I only come to the hospital. That’s one of the things he considers that and regulates my time out.”-PNC 3*

### Distance relational agency

Finally, social-cultural, and health systems factors presented various influences and circumstances that affected women’s ability to exercise agency and make choices in their lives. In this context, religious norms limited women’s access to evidence-based maternal care. Some respondents acknowledged that some religions challenged the idea of seeking healthcare in health facilities.

*“In terms of decision-making, the church does not hinder anyone, but our church believes in not taking medication. We believe that if I am sick and you come to pray for me, I will be healed. However, it also says that if you know that your faith is not strong enough, you can go and seek medical treatment. But after you have received treatment, don’t come back to the church until you have finished whatever you need to do. Then, you can call on the elders to come and pray for you before returning to the church.”-CHV*

Patriarchal structures including male dominance also limited women’s ability to exercise agency and make decisions independently. Most respondents emphasized men’s cultural role in decision-making regarding seeking MCH care. They acknowledged that increasing the engagement of men would yield considerable health benefits and provide an important avenue for giving men information which would also foster trust between spouses.

*“Well, I would really wish to bring him along so he can see for himself how busy it is, from here to there. If he doesn’t see it for himself, he won’t understand.”-PNC 3*

However, there were instances where male involvement was recognized as enabler for agency, as evidenced by men accompanying their spouses for clinics.

*“You see, for instance, today my husband accompanied me, and he needed to go to work, so he brought me and went to work, and he finished what he was doing, and now he is calling me because we have been waiting here for a long time.”-PNC 1*

Additionally, gender norms affected women’s ability to make decisions at the household level. Several women had partial control of their households’ finances but also kept financial secrets from their partners. This could be attributed to the feeling of being disempowered and the belief that financial secrets would give them a sense of autonomy. Interestingly, even after saving this money secretly, some women still needed their spouse’s approval to spend.

*“They (women) also fear, even after saving they still can’t use the money without getting approval from the husband because they cannot do anything. It is mandatory, the husband has to know.”-MCH in charge*

Women also stressed the need for securing the buy-in of the decision makers through community units, the nyumba kumi initiative, counselors, and healthcare providers.

*“If we can find partners like you, we can go to the assistant chief’s council through the community health volunteers (CHVs). We can invite men to attend, and even if not all of them will come, we can create a network by telling a friend who tells another friend. This is like politics, but if we can use this network to spread the importance of maternal healthcare, it will be beneficial.”-CHV.*

Health system factors such as facility workload, wait times, staff availability, and distance to health facilities also impacted women’s agency. The healthcare facilities were often overwhelmed with a high patient load, leading to longer waiting times, and delays in receiving care which can reduce women’s agency in seeking timely healthcare and may force them to delay or forgo necessary care. Women’s ability to make decisions about their own health was compromised when they had limited choices or faced barriers to accessing services. It also created a burden on women who had additional responsibilities, such as caregiving or work, as they struggled to find time to visit busy healthcare facilities.

*“The health workers here are often overworked and have many patients to attend to. This leads to longer waiting times, which can sometimes make the patients frustrated.”-CHV*

*“Because of the staff shortage, we have only 2 staff in the ANC area. So, if one goes on leave, the one who is there serves both sides, the ANC and the other clients, the immunization side. So, the long queues and the long waiting time affect them, they don’t like it. I think that demotivates them even just to come to seek the services” -NO IC.*

The distance between women’s residences and healthcare facilities also impacted their agency due to challenges related to transportation, time constraints, and financial resources required for travel.

*“Most of them I’ve seen prefer the rural facilities, that is health centers or dispensaries instead of coming back to Lumakanda. I think the issue affecting them is transport.”-MS.*

*“At night, for a car, you’ll spend Ksh. 2,000 and if it’s a motorcycle, you’ll give him Ksh.800 shilling.”-FGD-BC*

In some cases, where improved road networks existed, there were reports of women being able to access hospitals easily without difficulties.

*“They come to Lumakanda because the road is tarmacked, so, if it’s a pregnant woman, she will not be spun in potholes.”-FGD Men*

Our results indicate that individual agency, immediate relational agency, and distance relational agency influence women’s agency and autonomy. In terms of individual agency, factors such as self-stigma, trust in oneself, social networks, and previous birthing experiences emerged as important determinants of women’s decision-making power and control over their lives. Immediate relational agency, on the other hand, highlighted the significance of household decision-making dynamics, including aspects like control of household finances, respect between couples, and spousal communication. These factors played a crucial role in shaping women’s ability to exercise agency within their immediate relationships. Furthermore, distance relational agency demonstrated the impact of health system factors and socio-cultural norms on women’s agency and autonomy. Factors such as facility workload, staff availability, and religious norms were found to significantly influence women’s decision-making power and freedom of movement. Overall, these findings underscore the importance of recognizing and addressing various dimensions of agency and autonomy in order to empower women and promote gender equality.

## Discussion

Our work highlights a myriad of factors that influenced decision-making among women attending ANC, delivery, and PNC clinics during the early days of implementing a service delivery reform aimed at shifting all delivery to hospitals in a rural county in Kenya. Women’s autonomy and agency is still limited despite it being a crucial determinant of the use of maternal healthcare services. In our case, literacy was a pointer when analyzing women’s individual agency and decision-making capacity in engaging with MCH interventions and reforms. Our work revealed how, women with limited health literacy were unaware of their options in regarding participation in decision-making over their own reproductive health. Respondents reported the positive contribution of mass media in enhancing the household decision-making capacity. Similarly, Seidu et al. [33] demonstrated that women who watched television almost every day had a higher capacity to take household decisions, compared to those who did not watch television at [33] all. Mass media influences women empowerment, including their ability to take household decisions [34] by changing some socio-cultural norms such as gender stereotyping [35] suggesting that interventions to promote shared decision-making may be particularly important for patients with limited health literacy.

When women had individual agency, they reported having informed and better financial choices for themselves, and their families. Thus, individual agency elevated perceived self-efficacy in their decision making. Choices about individual women’s reproductive pathways and decision-making for care seeking therefore depended their perception of self; the self in relation to social environment and reflection on risks associated with the decision to seek care or not [36]. Social identity influences decision-making practices of individuals emphasizing the importance of deliberately embracing diversity and promoting inclusion future in the design of interventions and reforms with a focus on. This involves acknowledging and valuing different social identities, creating spaces that are safe and accessible for all individuals, and actively involving them in decision-making processes [37].

Women’s autonomy can also be better understood from a relational perspective since individual autonomy often fails to incorporate social reality. Relational autonomy posits that persons are socially embedded and that their identities are formed within the context of social relationships and shaped by a complex of intersecting social determinants and health system determinants [38]. We found that in both joint and nuclear families, women who have better spousal communication with their husbands have greater agency. The role of immediate relational agency is therefore mediated through the family context and the quality of relationships the women have which consequently influences their agency. Male heads of households were central in health decisions, and in some instances discussing health issues with their wives before final decisions were made [39, 40]. While joint and constructive communication leads to psychological well-being and protect against stressors during pregnancy [41] the process for decision-making often becomes delayed, consequently mothers’ ability to receive professional health care and other obstetric interventions on time.

Freedom of movement is also an important determinant of immediate relational agency, as the household structure affects women’s freedom of movement, since women residing in joint households are less likely to have decision-making power and need permission more often from other household members to execute some routine household activities [42]. Women require the permission of a husband or another male to pursue activities outside the home due to trust issues, social norms, and religious norm. This subsequently limited women’s ability to access and use skilled maternal health services including attending antenatal clinics or giving birth at a health facility. However, women who worked outside the home were more mobile, and women who were independent in the social sphere were also confident in their ability to negotiate independent mobility [40]. Additionally, it has been shown that husbands’ out-migration promotes women’s freedom of movement [43].

In summary, there is need for deliberate efforts towards empowering women’s autonomy in reproductive matters. Men might also benefit in the empowerment process through enlightenment and through effective implementation of male engagement interventions that leverage men’s power within households and promote women’s autonomy in decision making. Notably, women who receive male engagement education report making joint decisions (such as contraceptive choices, purchases of daily needs, and whether or not to work out of the home) compared to those who do not received such education [44].

The strength of this study includes the comprehensive exploration of agency across multiple dimensions, providing insights into the influence of factors such as previous birthing experiences, self-esteem, social support, financial control, respectful communication, and health system barriers on women’s agency. Although the challenge of establishing causality between the identified factors and women’s agency pose a challenge, the findings offer compelling descriptive explorations of the often undermined voice of women in shaping maternal and child health interventions policies and practices.

## Conclusions

The study findings underscore the limited autonomy of women in Kakamega County, emphasizing the importance of considering women’s decision-making in the successful implementation of the SDR. Moving forward, it is crucial for SDR implementation strategies to recognize and promote women’s autonomy, engaging decision-makers to understand the significance of women’s choices regarding delivery in higher-level facilities. This calls for a concerted effort to enhance women’s autonomy in reproductive healthcare through initiatives such as male involvement, women’s empowerment programs, access to resources, and institutional support. Additionally, MCH programs should prioritize health and financial literacy, freedom of movement, gender equality, and media access to counter cultural and religious barriers to women’s autonomy.

### Supplementary Information

**Supplementary Material 1.**

## Data Availability

The data that supports the findings of this study are available in the article and its supplementary material.

## Abbreviations

ANC: Antenatal clinics
CHVs: Community Health Volunteers
EmONC: Emergency obstetric and newborn care
FIF: Facility Improvement Fund
FGDs: Focus Group Discussions
ICRW: International Centre for Research on Women
IDIs: In-depth interviews
KII: Key Informant Interviews
NBU: Newborn Unit
NHIF: National Health Insurance Fund
SDG: Sustainable Development Goal
SDR: Service Delivery Redesign
