# Supplementary material for: Women’s autonomy and maternal health decision making in Kenya: implications for service delivery reform - a qualitative study
Source: BMC Womens Health. 2024 Mar 19;24:181. doi: 10.1186/s12905-024-02965-9 (PMC10949706; doi:10.1186/s12905-024-02965-9)
Supplement: Supplementary file 1 — Supplementary Material 1. [file 12905_2024_2965_MOESM1_ESM.docx]

**Supplementary file 1: Complexities of the Kakamega SDR**

|  | **SDR component** | **Intervention** | **Stakeholders involved.** |
| --- | --- | --- | --- |
|  |  |  |  |
| 1 | Infrastructural reorganization for quality care; and capacity to handle increased demand for Labour, delivery, and postnatal care | Construction of health facilities and strengthening diagnostic capacity, equipment, supplies, and medicines available | 1. **BuildX Studio** 2. Kakamega County government 3. Health system managers 4. Healthcare providers 5. Healthcare users |
| 2 | Emergency transportation/ referral | Emergency evacuation/referral services | 1. **Rescue. Co.** 2. County referral team 3. Local transportation authority 4. Transport unions 5. Local communications authority 6. Healthcare users |
| 3 | SMS prompts | SMS-based information to safely navigate and connect to the appropriate level of care in the event of an emergency | 1. **Jacaranda Health** 2. Kakamega County government 3. Health system managers 4. Healthcare providers 5. Healthcare users |
| 4 | Peer-to-peer mentorship program | The program trains providers to rapidly identify emergency cases in need of advanced care Emergency Medical Technicians (EMTs) and paramedics to provide better quality care en route to the hospital. | 1. **Jacaranda Health**. 2. Heath system managers 3. Healthcare providers |
| 5 | Digital blood tracker | The blood tracker provides instant visibility of supplies through its management tool and improves transport between blood satellite centers and facilities. | 1. **Jacaranda Health**. 2. **Regional blood bank** 3. Kakamega county government 4. Healthcare providers 5. Healthcare users |
| 6 | Health financing activities | Improve planning and budgeting processes and strategic purchasing through mobilization and advocacy activities at the county level. Training are also conducted at the facility level with the finance department on claims, priority setting and resource allocation. Technical assistance and support supervision is also conducted. | 1. **Think Well** 2. **Kakamega County**   **Government**   1. Heath system managers 2. Healthcare providers 3. Healthcare users |
| 7 | Human-centered design approaches | Introduction of ‘innovation labs; to prototype solutions, and the establishment of a Community of Practice for partners and the County to generate learnings and best practices for improving quality of care.  Introduced ticketing systems in the facilities for reduce long wait times at the ANC, PNC, and maternity. | 1. **Jacaranda Health** 2. **ThinkPlace** 3. Implementing partners 4. Healthcare providers 5. Healthcare users |
| 8 | Boosting Primary Care | Enhancing the quality of the suite of services provided at the primary care level including high-quality antenatal, postnatal, and child health services, and moving down basic NCD care to the health center and health post-levels | 1. **Kakamega County government** 2. **Heath system managers** 3. Healthcare providers 4. Healthcare users |
| 9 | Increasing provider numbers in hospitals | Transfer of nurses and clinical officers providing delivery and newborn care in lower-level facilities to hospitals. | 1. **Kakamega County government** 2. **County Public Service Board** 3. Health system managers 4. Healthcare providers |
| 10 | Strengthening Lifesaving care in hospitals | Careful monitoring of outcomes and rates of obstetric interventions, particularly Caesarean sections | 1. **Implementing partners** 2. **Heath system managers** 3. Healthcare providers 4. Healthcare users |
